# Physicochemical, Nutritional, and Organoleptic Characterization of a Skimmed Goat Milk Fermented with the Probiotic Strain *Lactobacillus plantarum* C4

**DOI:** 10.3390/nu10050633

**Published:** 2018-05-17

**Authors:** Miriam Moreno-Montoro, Miguel Navarro-Alarcón, Triana Bergillos-Meca, Rafael Giménez-Martínez, Silvia Sánchez-Hernández, Manuel Olalla-Herrera

**Affiliations:** Department of Nutrition and Food Chemistry, Faculty of Pharmacy, University of Granada, E-18071 Granada, Spain; mmorenom@ugr.es (M.M.-M.); bergillos@ugr.es (T.B.-M.); rafaelg@ugr.es (R.G.-M.); silsanchez@ugr.es (S.S.-H.); olalla@ugr.es (M.O.-H.)

**Keywords:** fermented goat milk, *Lactobacillus plantarum* C4, milk ultrafiltration, probiotic, physicochemical, nutritional and organoleptic characterization

## Abstract

The benefits of goat milk, fermented milks, and probiotics for the humans are well documented. In this study, a novel fermented goat milk was manufactured with the putative probiotic strain *Lactobacillus plantarum* C4 together with *L. bulgaricus* and *Streptococcus thermophilus*. Ultrafiltration was chosen as the skimmed milk concentration method because it produced the best viscosity and syneresis and a high casein content. The viability rate of all bacterial strains was >10^7^ cfu/mL, even after 5 weeks of storage or after in vitro gastrointestinal digestion, which is especially important for exertion of the probiotic strain functionalities. This fermented milk is also a good source of nutrients, having a low lactose and fat content, high protein proportion, and good mineral concentration. According to these data and the overall acceptability described by panelists, this fermented milk is a healthy dairy product comparable with commercially available fermented milks.

## 1. Introduction

There is increased interest in foods with a positive effect on health beyond their nutritional value, and considerable attention has focused on probiotic products. Fermented milks, especially when probiotics are present, have been attributed with numerous properties, including: an improvement in lactose absorption, increases in protein and fat digestibility and in antibacterial activity [1], immune system stimulation, preventive action against digestive system cancer, anticholesterolemic action, and the enhancement of mineral bioavailability, among others [2,3]. Consequently, fermented milk consumption has been recommended for lactose intolerance, diarrhea, constipation, *Helicobacter pylori* treatment, prevention/improvement of respiratory and gastrointestinal tract infections, strengthening of the immune system improvement, and atopic eczema, among other conditions [4,5]. In addition, pre- and pro-biotic-containing fermented infant formulae are frequently used to relieve or prevent symptoms of gastrointestinal discomfort in young infants [6].

Fermented milk is widely used to carry probiotic strains because the bacteria are kept alive, and its daily intake is recommended. However, cheese is in general the best carrier due to its buffer capacity and higher pH. There has been a long and safe history of dairy products containing the genus *Lactobacillus* [5]. Specifically, supplementation with *Lactobacillus plantarum* has been shown to have multiple benefits. Thus, long-term supplementation with *L. plantarum* TWK10 may increase muscle mass, enhance energy harvesting, promote health, enhance performance, and combat fatigue [7]. In another study, a significant decrease in blood pressure was observed in DOCA-salt hypertension-induced VaD rats after oral administration of *L. plantarum* TWK10-fermented soymilk extract [8]. The present investigation was designed to test the putative probiotic strain *L. plantarum* C4 in this context. This strain fulfills in vitro criteria for the selection of potentially effective probiotic bacteria and has demonstrated antimicrobial, microbiota-modulating and immunomodulating properties [9,10]. It could therefore be a highly valuable strain for the dairy industry and for healthcare. Skimmed milk was previously found to be an appropriate vehicle for the intragastric administration of this probiotic strain to mice [11]. 

In a previous study on the antioxidant, ACE-inhibitory and antimicrobial activity of fermented goat milks with the classical starter bacteria (St: *L. bulgaricus* and *Streptococcus thermophiles*) or with the St plus the *L. plantarum* C4 probiotic strain, no differences in biological activities were observed between the two fermented milks [1]. Only, some antimicrobial activity against *E. coli* was observed for the fermented milk containing the probiotic, probably be due to some peptides being released by the probiotic strain [1]. Additionally, in the in vitro evaluation of the fermentation properties and potential probiotic activity of *L. plantarum* C4 [10] it was concluded that this probiotic strain could modulate the intestinal microbiota in vitro, promoting changes in some numerically and metabolically relevant microbial populations and shifts in the production of small short fatty acids (SCFA) [10]. 

Goat milk has shown better digestibility, mineral bioavailability, and protein and fat profiles in comparison to cow milk [3], and the consumption of probiotic fermented goat milk has been attributed with certain therapeutic properties [12]. However, the production of goat set-yoghurts must overcome some technical challenges, because the low buffering capacity of goat milk increases the risk of over-acidification, and its low *α_s_*_1_-casein content and high casein micelle dispersion can lead to the formation of an almost semi-liquid gel [13]. The low firmness and high surface whey separation that can affect this type of yoghurt can be reduced by rigorous monitoring of the heat treatment, incubation temperature, and concentrations of fat, protein and total solids, among other measures [14,15,16]. Among different milk concentration methods, ultrafiltration mainly concentrates caseins and minerals bound to them [17]. In addition, because no additional heat treatment is required, the properties of the milk are preserved, unlike in other traditional methods such as powdered caseinates or skimmed milk addition.

One of the most typical sensorial characteristics of goat milk is the caprine flavor derived from the presence of short and medium chain fatty acids, which is generally considered a negative organoleptic attribute [3,18]. It is essential to reduce this flavor in fermented goat milk.

The objective of this study was to develop an appropriate procedure to manufacture a novel probiotic fermented goat milk (PFM) with good physicochemical, nutritional, and organoleptic properties, selecting and standardizing the milk concentration method, evaluating bacterial interactions and viability (*L. plantarum* C4, *L. bulcaricus* and *S. thermophilus*), and carrying out physicochemical, nutritional, and organoleptic analyses. 

## 2. Materials and Methods 

### 2.1. Raw and Skimmed Milks

Raw milk from Murciano–Granadina goats was collected and frozen in Granada over one year to avoid seasonal variations. The absence of β-lactam antibiotics (penicillins and cephalosporins), sulfamides, and tetracyclines was tested with milk TriSensor and Heatsensor kits (Unisensor, Liège, Belgium), incubating at 40 °C. The raw goat milk (RM) was skimmed in a skimming centrifuge (Suministros químicos Arroyo, Santander, Spain) at 30–35 °C and then pasteurized for 30 min at 80 °C, determining the efficacy of the process by measuring phosphatase (Phosphatesmo MI, Macherey-Nagel, Germany) and lactoperoxidase (Peroxtesmo MI, Macherey-Nagel) activities. In addition, the microbiological quality of the raw and pasteurized milk was evaluated by counting colony forming units (CFUs) of mesophilic aerobic microorganisms and testing the absence of *Enterobacteriaceae* in the pasteurized milk [10,11]. 

### 2.2. Standardization of Probiotic Fermented Goat Milk Manufacturing 

The milk concentration method, fermentation conditions, and bacterial interactions and viability were evaluated in order to standardize manufacture of this probiotic fermented goat milk. 

#### 2.2.1. Selection of the Concentration Method

**Figure 1:**
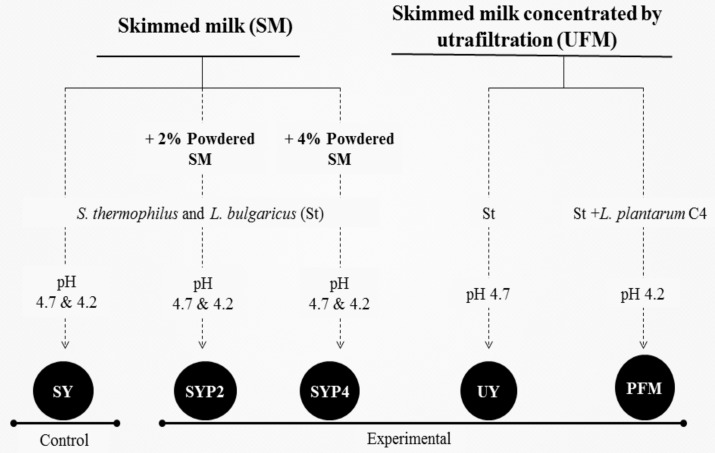
Selection of the concentration method. SY: Yoghurt made with skimmed milk (SM) fermented with the classical starter bacteria (St); SYP2: Yoghurt made with SM plus 2% of powdered SM fermented with St; SYP4: Yoghurt made with SM plus 4% of powdered SM fermented with St; UY: Yoghurt made with skimmed goat milk concentrated by ultrafiltration (UFM) fermented with St; PFM: Probiotic fermented milk made with UFM fermented with St plus *Lactobacillus plantarum* C4.

The influence of the milk concentration method on the fermented milk syneresis and viscosity was studied [19], comparing between the addition of powdered skimmed goat milk (2% or 4%) and ultrafiltration (Figure 1). The skimmed goat milk was concentrated by ultrafiltration as described by Bergillos-Meca et al. (2015) [20], filtering through a 50 kDa cut-off membrane (Vivaflow 2000, Sartorius Stedim) with a peristaltic pump (Masterflex^®^ L/S, Economy Drive, Cole Parmer^®^) at 2 bar pressure, up to a 12 ± 0.5% of dry extract, considered an adequate total solid content for the manufacture of fermented milks with a good consistency [21]. The following fermented milks were manufactured and analyzed: SY (made with skimmed milk: SM), SYP2 and SYP4 (with skimmed milk plus 2% and 4% of powdered skimmed milk, respectively), and UFY (with skimmed milk concentrated by ultrafiltration: UFM). The different milks were inoculated with classical freeze-dried starter bacteria (St), *L. bulgaricus* plus *S. thermophilus* (YO-MIX^®^ 350, Dupont™ Danisco, Barcelona, Spain) at the concentration recommended by the manufacturer. After inoculation, samples were distributed into sterile glass pots and incubated at 42 °C. The incubation was stopped at pH 4.7 or 4.2, being the usual pH in yoghurt manufacture and the isoelectric point of goat milk proteins, respectively. Samples were then stored under refrigeration at 4 °C. Three samples of each type of fermented milk were manufactured and analyzed in triplicate.

#### 2.2.2. Viability and Interactions of the Fermenting Bacteria 

Interactions among *L. bulgaricus*, *S.* *thermophiles* and *L. plantarum* C4 were investigated using the spot test on Tryptone Soy Agar (TSA) (Difco™, Becton, Dickinson and Company; Madrid, Spain), Man Rogosa Sharpe (MRS) agar (Difco™, Becton, Dickinson and Company; Madrid, Spain) and *L. plantarum* selective medium [22] (LPSM) agar plates, according to Bergillos-Meca et al. (2013a) [23].

The fermentation was also standardized in terms of time, temperature, and viable probiotic bacteria. For St, the inoculation was carried out as described above. For the inoculation of *L. plantarum* C4, the strain was recovered after overnight growth in MRS broth (Difco™, Becton, Dickinson and Company; Madrid, Spain) and re-suspended in the pasteurized milk after washing with sterile phosphate buffered saline (PBS; Sigma-Aldrich, Steinheim, Germany). 

First, the appropriate fermentation temperature was selected. The milk was fermented with St and *L. plantarum* at their known optimum growth temperature (42 °C for St and 37 °C for *L. plantarum*. 

Second, the fermentation time and probiotic bacteria count were standardized by manufacturing six different types of fermented goat milk: SM and UFM fermented for 8 h with St, *L. plantarum* C4 or St and *L. plantarum* C4 together at selected temperatures, inoculating the same concentrations of *L.* *plantarum* C4 and St (10^6^ cfu/mL). The microorganism count was done at different time points during the fermentation process by plating serial dilutions on TSA, MRS and LPSM agar and incubating for 24–48 h at 37 °C [10,11,23].

### 2.3. Analysis of the Standardized Probiotic Fermented Goat Milk

For this analysis, 8 batches of probiotic fermented goat milks were manufactured at different time periods weeks over one year and three samples of each batch were analyzed. 

All analyses were done in triplicate, and blanks were prepared and analyzed following the same procedures.

#### 2.3.1. Viable Bacteria after Fermentation and Storage

Viable microorganisms in the final PFM were counted by plating serial dilutions of the sample on TSA, MRS and LPSM agar plates and incubating for 24–48 h at 37 °C. In order to assay their viability during storage, they were counted weekly until 6 weeks after their manufacture.

#### 2.3.2. Viable Bacteria after In Vitro Gastrointestinal Digestion

Samples underwent simulated gastrointestinal digestion in duplicate as described by Bergillos-Meca et al., 2013b [24]. Briefly, 20 g of each fermented milk were homogenized with 80 mL of bidistilled deionized water. For gastric digestion the pH was adjusted to 2.0 with 6 M HCl. The pH was checked after 15 min and if necessary readjusted to 2.0, then an amount of freshly prepared pepsin solution, sufficient to yield 0.02 g pepsin/sample, was added. The sample was incubated in a shaking water bath at 37 °C and 120 strokes/min for 2 h. The dialysis assay comprised the pervious gastric step followed by an intestinal step where dialysis was included (dialysis bag: molecular weight 12–14 kDa; Visking 45 mm × 27 mm, Medicell International, London, UK). Dialysis tubing, containing 25 mL of bidistilled deionized water and an amount of NaHCO_3_ equivalent to titratable acidity measured previously, were placed in the flasks together with 20-g aliquots of the pepsin digest and incubated in the shaken bath at 37 °C for 30 min. An amount of freshly prepared pancreatin-bile extract mixture (0.001 g pancreatin and 0.006 g bile salts/samples) was added to the flask and the incubation continued up to 2 h. Dialyzable and non-dialyzable fractions were weighted, freeze dried, and stored until the assay [24]. 

After gastric and intestinal steps, serial dilutions of the digested product were plated on LPSM and MRS to count viable microorganisms [10,11,23].

#### 2.3.3. Viscosity 

The viscosity was determined using a Brookfield DV-II + Viscosimeter (Brookfield, Harlow, UK) equipped with a 21-spindle following a previously reported procedure [19]. The sample stress was measured in the PFMs and a commercial probiotic skimmed fermented goat milk (GFM; with St and a probiotic *Bifidobacterium* strain) using different rpm values at 20 °C.

Fermented goat milk samples were also distributed into 100 mL volumetric cylinders for weekly measurement during one month of the volume of whey separated from the curd, as described by Moreno-Montoro et al. (2013) [19]. The sample stress was measured in the manufactured fermented milks and a commercial probiotic skimmed fermented goat milk (with St and a probiotic *Bifidobacterium* strain) using different rpm values at 20 °C.

#### 2.3.4. Spontaneous Syneresis 

Fermented goat milk samples were also distributed into 100 mL volumetric cylinders for weekly measurement during one month of the volume of whey separated from the curd, as described by Moreno-Montoro et al. (2013) [19].

#### 2.3.5. pH and Acidity 

The pH and acidity of the fermented goat milks were measured according to the Association of Official Analytical Chemists (AOAC, 2006) procedure [25]. 

#### 2.3.6. d/l-Lactic Acid Test

d- and l-lactic acid isomers were measured with the Boeringer Mannheim d-Lactic/l-lactic test (R-Biopharm, Darmstadt, Germany) using a certified standard supplied in the kit for the validation; recovery of 99.7% was obtained. 

#### 2.3.7. Dry Extract

The dry extract content was measured according to the AOAC method (2006) [25]. 

#### 2.3.8. Lactose and Galactose 

Lactose and *D*-galactose levels were measured with the Megazyme enzymatic kit (Wicklow, Ireland), using a certified standard supplied in the kit for the validation; recovery of 98% was obtained. 

#### 2.3.9. Proteins 

Total protein concentration was measured by the Kjeldahl method according to Olalla et al. (2009) [26] but weighing 3 g of fermented milk. Bovine serum albumin (Sigma-Aldrich, Steinheim, Germany) was used for the validation; recovery of 97.5% was obtained.

#### 2.3.10. Fat 

Fat concentration was determined using the Gerber method adapted for fermented milks according to the AOAC method (2006) [25].

### 2.4. Sensorial Analysis

Three samples were selected for analysis by ten trained judges: (1) PFM; (2) GFM; and (3) CFM: Commercial skimmed cow yoghurt fermented with St. The screening test was interpreted according to ASTM [27]. In each season, four tablespoons of each sample were presented to the panelists in plastic plates that were randomly coded using three-digit numbers and two letters. Table 1 exhibits the response form designed for this study and the parameters analyzed [3,28,29,30,31].

### 2.5. Statistical Analysis 

The homogeneity of variance was assessed using the Levene test and the normal distribution of the samples with the Shapiro-Wilk test. The Student’s *t*-test was used to analyze parametric data and the Kruskall–Wallis test to analyze non-parametric data. Finally, the relationship between assays was evaluated by computing the correlation coefficient by Pearson linear correlation (for normally distributed variables) or Spearman linear correlation (for non-normally distributed variables). The significance level was set at 5% (*p* < 0.05) in all tests. SPSS 15.0 for Windows (IBM SPSS Inc., Chicago, IL, USA) was used for data analyses.

## 3. Results and Discussion

### 3.1. Standardization of Probiotic Fermented Goat Milk Manufacture 

#### 3.1.1. Selection of Concentration Method 

The physical properties of set-yoghurt are very important for consumer acceptance. Spontaneous syneresis is the contraction of a gel with no external force application and is related to instability of the gel network and consequent loss of the ability to entrap all of the serum phase [14]. There is no standardized method for its quantification, hampering the comparison of results. Among various factors related to viscosity and syneresis in fermented milks, we focused on the concentrations of protein and solids.

In summary, as detailed below (in Section 3.2.3 and Section 3.2.4), ultrafiltration proved to be superior to powdered milk addition as a goat milk concentration method, with increased whey retention in the final product and better viscosity. 

#### 3.1.2. Viability and Interactions of the Fermenting Bacteria 

Considerable research attention is being paid to the selection of bacterial strains for efficient fermentation [32], *L. plantarum* C4 was the only strain showing no change in growth in the spot test on MRS agar, while inhibition was only observed on *L. bulgaricus* by *L. plantarum* C4 in the test on TSA, which may be due to the acid production from the high dextrose concentration in MRS. The action of *L. plantarum* C4 on *L. bulgaricus* in TSA may be attributable to bacteriocin-like substances produced by *L. plantarum* C4. Nevertheless, when the three strains were co-cultured in goat milk, St growth was not inhibited by *L. plantarum* C4. One explanation for the difference may be that microorganisms use dextrose in MRS but lactose in milk. These data underscore the importance of environmental factors, such as the nature and concentration of sugars. Thus, it is well documented that the growth and fermentation rates of probiotics depend on the carbohydrates present during fermentation [33]. 

With respect to the fermentation temperature, whereas the probiotic bacteria *L. plantarum* C4 showed hardly any growth at 42 °C, they grew by almost one exponential unit at 37 °C. On the other hand, the growth of starter cultures was not affected by the fermentation temperature. The fermentation temperature was therefore set at 37 °C to obtain the maximum concentration of probiotic bacteria in the final product.

When *L. plantarum* C4 was used alone, the growth was higher but no change in pH was obtained; therefore, it was used together with St to obtain the fermented milk. Although some interaction has been described between probiotic strains and St microorganisms [32], growth of both microorganisms present in St grew to the same degree during the milk fermentation when used alone and when used with the probiotic strain, as previously demonstrated for the probiotic bacteria *L. helveticus* R0050 [33]. This exemplifies the variability in relationships and interactions between St and probiotics, which require further research to determine whether they result from substrate competition, the production of inhibitory compounds, proteolysis, or simply from the changes in pH during fermentation. When used together with St, *L. plantarum* C4 grew ~0.90 Log cfu/mL at 6 h, and its growth was not influenced by the milk used, reaching around 6.75 Log units. Therefore, in order to obtain a high load of viable probiotics in the final fermented milk, the initial probiotic concentration in the milk was 10^8^–10^9^ cfu/mL, high enough to exert its healthy properties. No negative interactions were observed among the fermenting strains.

Based on the above findings, the optimum fermentation conditions were inoculation of the probiotic strain at 10^9^ cfu/mL followed by fermentation at 37 °C for around 6 h, when pH 4.2 is reached. 

Once fermentation conditions were optimized, the following manufacturing process was carried out: after UFM pasteurization, milk was quickly cooled and then inoculated with St plus *L. plantarum* C4, obtaining PFM. The inoculated milk was distributed in sterile 200 mL glass pots and incubated at 37 °C up to a pH of 4.2. Finally, the fermented milks were rapidly cooled and stored at 4 °C. 

### 3.2. Analysis of the Standardized Probiotic Fermented Goat Milk

#### 3.2.1. Viable Bacteria after Fermentation and Storage

The mean concentration ± SD of viable bacteria in PFM was 8.98 ± 0.32 Log cfu/mL for *L. plantarum* C4 and 8.72 ± 0.31 Log cfu/mL for St. Concentrations always exceeded 10^7^ cfu/mL, the minimum required to manufacture fermented milk, and were within the reported range for starter cultures [34]. 

**Figure 2:**
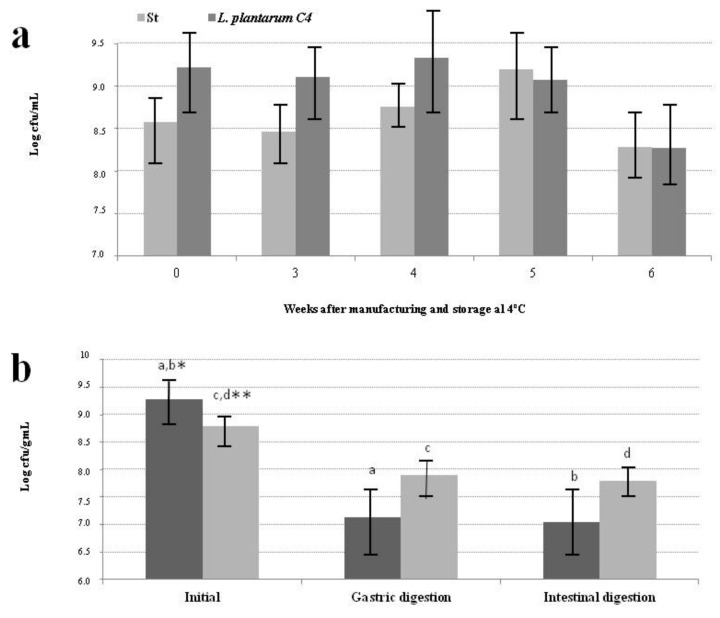
Number of viable bacteria in the probiotic fermented goat milk (PFM) manufactured with skimmed milk concentrated by ultrafiltration and fermented with St (classical starter bacteria) and *Lactobacillus plantarum* C4 by the standardized procedure. (**a**) During the storage at 4 °C; (**b**) After in vitro gastrointestinal digestion. St: classical starter bacteria (*L. bulgaricus* plus *S. thermophilus*). ^a,b,c,d^ Mean values ± SD with the same superscript denotes the existence of significant differences: * *p* < 0.001, ** *p* < 0.05.

A non significant increase was observed in all viable bacteria up to 4 weeks of storage at 4 °C. A non significant slow decrease began in *L. plantarum* C4 at week 4 and in St at week 5 (Figure 2a). Nevertheless, the percentage viability was higher than 10^7^ cfu/mL after 6 weeks of cold storage. 

#### 3.2.2. Viable Bacteria after In Vitro Gastrointestinal Digestion 

In vitro gastric digestion produced a significant fall (*p* < 0.001) of almost one Log unit in the *L. plantarum* C4 count and of more than two Log units in the St count (Figure 2b). However, St and *L. plantarum* C4 showed a similar resistance to in vitro intestinal digestion. Thus, the concentration of viable *L. plantarum* C4 was around 10^8^ cfu/mL after in vitro gastric digestion, a resistance of 90%, which is within the range recommended by some authors for a probiotic strain to exert its function and deliver its benefits to the consumer [35]. With regard to the starter bacteria, resistance to low pH of between 0 and 48% has been reported for *L. bulgaricus* and between 0 and 45% for *S. thermophilus* [36], lower than observed for St in the present study (Figure 2b). With respect to the intestinal stage, despite high resistance to bile salts for *L. plantarum* C4, medium resistance has been reported for *S. thermophilus* and high vulnerability for *L. bulgaricus*, while all of the bacteria showed high resistance (99%) in our study [11,36]. This difference could be due to a potential matrix protective effect, differences among strains, and/or interactions due to their joint presence in PFM, which requires evaluation in future studies. 

In summary, the viability of all bacteria was maintained at an acceptable concentration during fermentation and after 5 weeks of storage. They also proved to be resistant to in vitro gastrointestinal digestion.

#### 3.2.3. Viscosity

**Figure 3:**
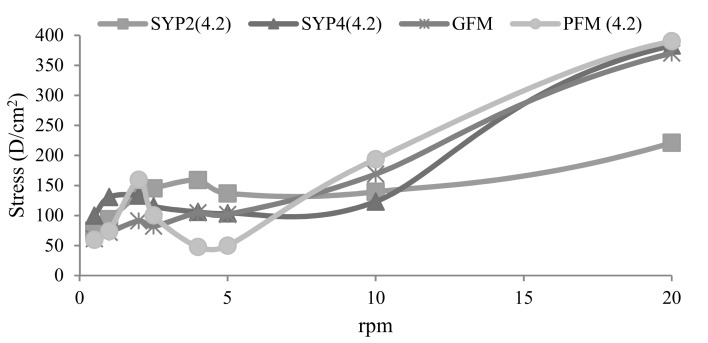
Viscosity curves of the goat milks fermented until pH 4.2, compared with a commercial one. SYP2 and SYP4: Yoghurt made with skimmed milk (SM) and 2% or 4%, respectively, of commercial powdered SM, fermented with the classical starter bacteria (St); GFM: Commercial probiotic skimmed fermented goat milk (St and *Bifidobacterium*); PFM: Probiotic fermented goat milk made with skimmed milk concentrated by ultrafiltration, fermented with St and *L. plantarum* C4.

The best viscosity was observed for UFY and the other fermented milks when fermentation was stopped at pH 4.2. Therefore, the pH 4.2 was chosen for stopping fermentation. Specifically, the viscosity of PFM and SYP4 (skimmed goat milk + 4% of powdered skimmed goat milk) was similar to that of the GFM and significantly different (*p <* 0.05) from that of SYP2 (skimmed fermented goat milk + 2% of powered skimmed goat milk) (Figure 3). 

#### 3.2.4. Syneresis

**Figure 4:**
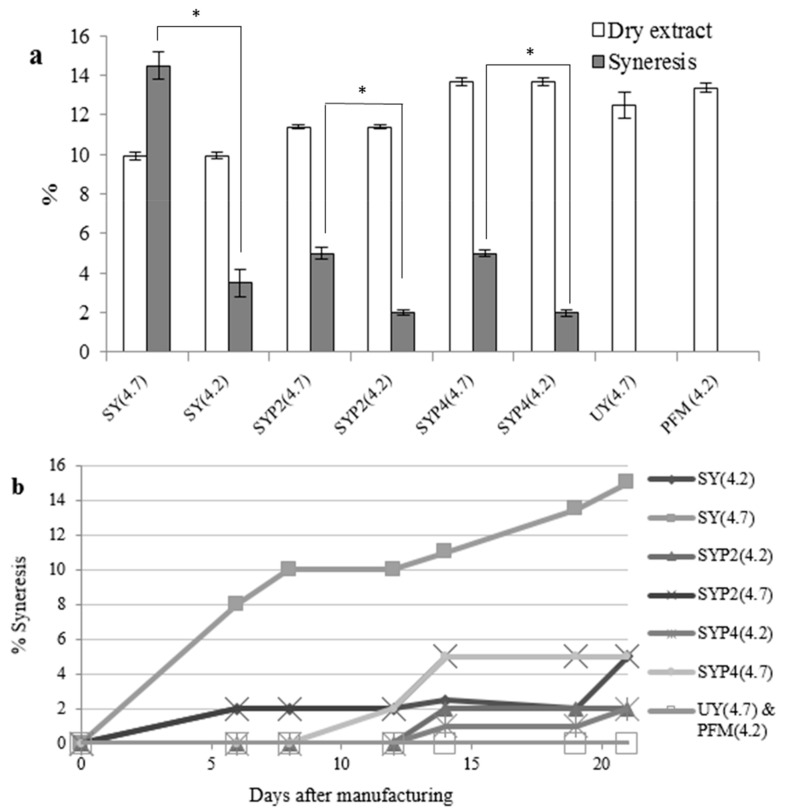
(**a**) Dry extract content and syneresis after 21 days of storage at 4 °C; (**b**) Syneresis evolution over 21 days of storage in fermented milks concentrated by different methods. SY(4.2) and SY(4.7): Yoghurt made with skimmed milk (SM) fermented with the classical starter bacteria (St) when the fermentation was stopped at pH 4.2 or 4.7, respectively; SYP2(4.2) and SYP2(4.7): Yoghurt made with SM plus 2% of powdered SM fermented with St when the fermentation was stopped at pH 4.2 or 4.7, respectively; SYP4(4.2) and SYPA(4.7): Yoghurt made with SM plus 4% of powdered SM fermented with St when the fermentation was stopped at pH 4.2 or 4.7, respectively; UY(4.7): Yoghurt made with skimmed goat milk concentrated by ultrafiltration (UFM) fermented with St when the fermentation was stopped at pH 4.7; PFM(4.2): Probiotic fermented milk made with UFM fermented with St plus *L. plantarum* C4 when the fermentation was stopped at pH 4.2. In figure (**a**), significant differences between the syneresis of each sample at pH 4.7 and 4.2 were signaled as follows: * *p* < 0.001.

Syneresis diminished with addition of greater dry extract and with ultrafiltration (Figure 4a). Improvement in rheological behavior with higher solid content has previously been reported [15]. In addition, when the final pH was 4.2, a lower final syneresis was found (Figure 4b). However, the best whey retention was observed when ultrafiltered milk was used, with no significant variation at different pH values (Figure 4a). The addition of powdered skimmed milk led to similar viscosity but greater syneresis in comparison to ultrafiltration. In this regard, distinct rheology properties have been described for fermented milks concentrated by different methods [37]. These differences may be attributable to casein alteration by the spray-drying process or to the increase of all milk compounds in the same proportion with the addition of powdered skimmed milk, whereas ultrafiltration of milk mainly concentrates caseins, which are responsible for formation of the yoghurt coagula [17,38]. Syneresis in fermented milks may be dependent on the pH because the isoelectric point of caprin caseins (at which they start to aggregate) is reached at pH 4.2, forming the gel network. At pH 4.7, the isoelectric point of cow caseins, there is a lesser aggregation of caseins, forming a gel network with a higher tendency to syneresis [16].

PFM showed a very low mean syneresis value (0.20 ± 0.25%), denoting good coagula formation with adequate whey retention. In this regard, there is a lesser tendency to syneresis when the water is better retained in the protein network. However, it has been reported that other parameters are also important in this process, such as total solid and protein content, concentrations of Ca^2^^+^ and fat, pH, fermentation temperature, and preheating of the milk [38]. Thus, Bergillos-Meca et al. (2015) [20] demonstrated higher Ca, P, Mg and Zn concentrations in fermented goat milks manufactured with ultrafiltration-concentrated milk, which could favor the improved gel formation in PFM.

The addition of *L. plantarum* C4 is also of interest because it did not change the textural properties of the final product and could contribute to a healthier final product due to its probiotic effects, including the angiotensin-I-converting-enzyme inhibitory and antioxidant activities reported for the small and non-basic bioactive peptides in this PFM [1,39].

#### 3.2.5. pH and Acidity

Table 2 displays the pH and acidity values in the final product. Although the pH of the PFM was in the range of reported values for fermented goat milks (from 3.83 to 4.32 g lactic acid/100 g), the acidity was slightly higher (from 0.876 to 1.08 lactic acid/100 g) [40,41]. This may be explained by the increased protein concentration, given reports that the titratable acidity content in yoghurts is influenced by the type of protein used to fortify the total solid content of the milk base [37]. 

#### 3.2.6. d/l-Lactic Acid Levels 

According to Beal et al. (1999) [34], lactic acidification by starter strains is influenced by the quality of the milk, the strains used, and the incubation temperature. They reported that the post-acidification process also contributes to the acidity and is mainly affected by the strains used and the storage temperature and duration. In our samples, although *L. plantarum* C4 proved to be lactose-positive, however as previously described, no decrease in pH was observed after fermentation.

In relation to lactic acid isomers, d-lactic is produced by *L. bulgaricus* and is attributable to the post-acidification, whereas l-lactic is produced by *S. thermophilus* and is usually more concentrated in non-stored fermented milks [34]. Therefore, the proportion of these isomers depends on the intensity of fermentation by the bacteria and on the storage duration and conditions. The percentage of l/d-lactic acid in PFM was 57/43, indicating that both St bacteria have similar fermenting activity. These values are within the range of 55/45 to 60/40 reported by Kneifel, Jaros and Erhard (1993) [42] for five fermented cow milks manufactured with commercial strains. The same authors observed a wide variability, although the l-lactic concentration was generally higher, as observed in the present samples (Table 2). Other authors reported different amounts of these isomers, from 0.13 to 0.6 g l-lactic acid/100 g and from 0.5 to 0.93 g d-lactic acid/100 g [41,43].

#### 3.2.7. Dry Extract Content

This parameter mainly depends on the fat, protein and carbohydrates contents of the milk and therefore varies widely according to the milk used. The dry extract of UFM (Table 2 [20]: 11.5 ± 0.3 g/100 g) was previously selected because it achieved good textural properties and is in the level of other fermented milks. Reported values ranged from 9.88 g/100 g [41] with the utilization of skimmed milk to 17.8 g/100 g with previously concentrated whole milk [13].

#### 3.2.8. Lactose and Galactose Levels 

The lactose content is one of the main factors responsible for curd formation, because it determines the acidification by lactic acid production. The lactose concentration used for fermentation in UFM was 4.92 g/100 g, which was sufficient to obtain a good curd according to those findings. There was a higher percentage of lactose in PFM (2.44 ± 0.60 g/100 g; Table 2) than reported in other fermented goat milks (between 1.19 and 1.8 g/100 g) [13,41], although concentrations in fermented cow milks reach up to 4.84 g/100 g [42]. These differences may be attributable to the fermenting bacteria used, the fermentation process, or variations in the lactose concentration of the milk, which is higher in cow *versus* goat milks [18]. There was only a small concentration of galactose (0.42 ± 0.14; Table 2), one of the end products of milk fermentation, whereas other authors reported concentrations up to 1.64 g/100 g [41], which may be because this carbohydrate is metabolized by *L. plantarum* C4, which is galactose-positive. Similar percentage lactose and galactose values to the present findings were described by Quintana López (2011) [41] in fermented cow milks and kefir (lactose: 2.82 and 2.97 g/100 g; galactose: 0.68 and 0.37 g/100 g; for fermented cow milks and kefir, respectively). These data support the proposition that these discrepancies may be explained by the different fermenting strains used, as previously reported [42]. 

#### 3.2.9. Protein Concentration

The protein concentration in PFM was much higher than reported for other fermented goat milks (from 3.29 to 3.99%) [18,41] because ultrafiltration was used. However, it was within the range found in fermented milks in which proteins were concentrated by different methods (protein values up to 6.65%) [21,44]. 

Given the high protein concentration of PFM, the better characteristics of proteins in goat *versus* cow milks, and the ultrafiltration process, which avoids heating-induced protein alterations, PFM can be considered a superior source of proteins in comparison with other reported fermented milks. 

#### 3.2.10. Fat Concentration 

The fat concentration in PFM was lower than the detection limit of the method (Table 2) because the goat milk used was skimmed, allowing it to be sold in sections for light, 0%, or healthy products.

#### 3.2.11. Sensorial Analysis 

**Figure 5:**
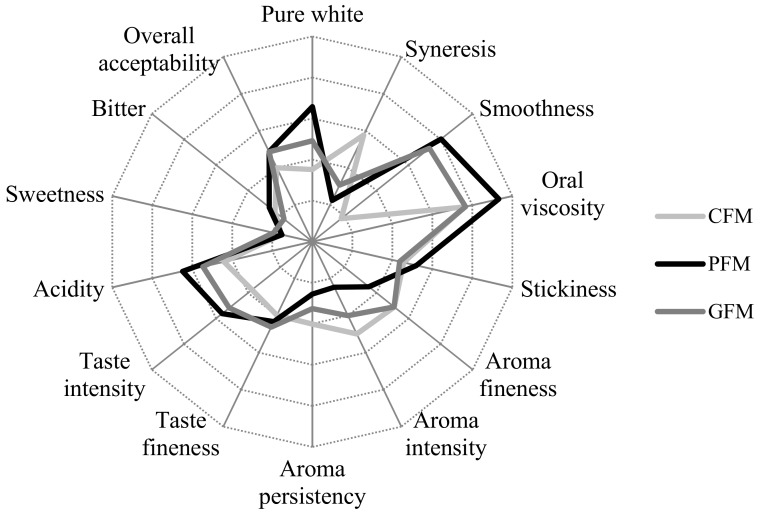
Representation of the quantitative sensorial parameters of analyzed fermented milks. CFM: Commercial skimmed cow yoghurt fermented with the classical starter bacteria (St); PFM: Probiotic fermented goat milk manufactured with ultrafiltered skimmed milk fermented with St plus *L. plantarum* C4; GFM: Commercial skimmed fermented goat milk fermented with St and a strain of *Bifidobacterium*.

Figure 5 depicts the sensorial profile of the fermented milks analyzed. Overall, the sensorial profile was similar for the fermented goat milks but different for commercial skimmed fermented cow milk (CFM). Consumers first perceive the color of fermented milk, and the white color was purest in PFM and least pure in CFM, probably due to the higher concentration of vitamin A in cow milk and the resulting light-yellow hue. Variations among the fermented goat milks can derive from differences in the fermentation process, which was found to influence the color of set-style yoghurts [45]. A curd formed by large grains with high syneresis is a negative characteristic that could lead to consumer rejection [46]. The importance of pH on the viscosity and syneresis has previously been observed. Panelists recorded the lowest syneresis in PFM and the highest in CFM, which was found to have the lowest smoothness. In addition, although lumps were observed in all samples, panelists described the curd of PFM and GFM as homogeneous, with CFM being the least homogeneous and receiving negative scores for all visual parameters. Hence, PFM had the best textural properties, although statistical significance (*p <* 0.05) was only reached for the difference in viscosity versus the other samples. 

Consumer preferences are also influenced by the vapor phase odor, which is first perceived when the yogurt pot is opened. CFM was considered to have the best aroma, which may be attributable not only to the different milk but also to the strain used and the fermentation and storage conditions. The aroma of yoghurt is mainly produced by acetaldehyde, which was perceived less in PFM than in the other samples. Kavas et al. (2003) [47] also observed a lack of acetaldehyde flavor in fermented goat milks concentrated by ultrafiltration. 

Taste parameter findings were better for PFM and GFM than for CFM, despite their similar perceived taste fineness, due to their high intensity. The goat flavor is considered a negative quality, and some researchers have recommended its elimination to enhance consumer preference in comparison to fermented cow by-products [3]. In the present study, most but not all of the panelists perceived a goat taste in the two fermented goat milks, indicating that the goat taste intensity was not high. Given that short chain fatty acids are responsible for this taste, it was probably reduced in PFM by the skimming process. Its overall acceptability was most influenced by the taste fineness (*p <* 0.001; r^2^ = 0.747), which was weakly correlated with aroma fineness (*p <* 0.05; r^2^ = 0.337). The taste fineness showed a weak positive correlation with the sweetness (*p <* 0.01; r^2^ = 0.409) and a weak negative correlation with acidity (*p <* 0.05; r^2^ = −0.399), consistent with previous studies [14]. The only defect reported was the insipid taste of CFM.

Finally, despite variations in sensorial profile, there were no significant differences in overall acceptability, indicating that all products (PFM, GFM and CFM) would be accepted by customers to a similar degree.

To summarize, the PFM was found to have a good appearance and texture, with only a few lumps. This fermented goat milk does not have a strong aroma and achieved overall acceptability rates within the range observed for the commercial fermented milks analyzed, despite its high acidity.

## 4. Conclusions

This novel fermented milk, manufactured with ultrafiltration-concentrated skimmed goat milk, has low lactose and fat concentrations, high protein proportion and mineral content, and good sensorial properties. Hence, PFM is an excellent source of nutrients, low in calories, and potentially functional due to the probiotic bacteria it contains. The viability rate of all bacterial strains was >10^7^ cfu/mL, even after in vitro gastrointestinal digestion. Together with the description by panelists of its overall acceptability, these properties allow PFM to be considered a good dairy product, comparable with commercially available fermented milks.

## Figures and Tables

**Table 1:** Parameters analyzed in the response form.

	Evaluation	Score	Parameter	Description
Visual	Scale of perception	0–5	Color	From grey/yellow-white to pure white
			Syneresis	Amount of water on the sample surface
			Smoothness	Smooth appearance, free of irregularities
	Presence/absence		Curd homogeneity	Fermented milk resembles flour
			Floury	
			Lumps	
			Bubbles	
Texture	Scale of perception	1–8	Oral viscosity	Yoghurt resistance to flow in mouth
		1–4	Stickiness	Degree to which the sample sticks or adheres to teeth and palate
Aroma	Scale of perception	0–5	Aroma fineness	Natural yoghurt-like aroma
			Aroma intensity	Perceived strength of the aroma
			Aroma persistency	Perceived duration of the aroma
	Presence/absence		Acetaldehyde	Yoghurt-like aroma
			Diacetyl	Butter-like aroma
			Goat boiled milk	
Taste	Scale of perception	0–5	Taste fineness	Natural yoghurt-like taste
			Taste intensity	How strong the taste is perceived
			Sweetness	Sweet taste
			Acidity	Acid taste
			Bitterness	Bitter taste
	Presence/absence		Goat, astringent, metallic, salty, insipid, boiled milk	
Overall acceptability				Final impression of the yoghurt

**Table 2:** Physicochemical and nutritional parameters measured in the developed probiotic fermented goat milk.

Parameter	Mean ± SD	Parameter	Mean ± SD
pH	4.19 ± 0.23	Galactose (g/100 g)	0.42 ± 0.14
Total acidity (g lactic acid/100 g)	1.09 ± 0.18	Proteins (g/100 g)	5.83 ± 0.13
d-Lactic acid (g/100 g)	0.368 ± 0.113	Fat (g/100 g)	<0.1
l-Lactic acid (g/100 g)	0.493 ± 0.154	Ca (mg/100 g)	164 ± 3.31 ^a^
Syneresis (g/100 g)	0.20 ± 0.25	P (mg/100 g)	84.4 ± 7.29 ^a^
Dry extract (g/100 g)	11.5 ± 0.3	Mg (mg/100 g)	15.6 ± 0.325 ^a^
Lactose (g/100 g)	2.44 ± 0.60	Zn (mg/100 g)	0.588 ± 0.040 ^a^

^a^ from Moreno-Montoro et al. (2015) [17].
